# Long-Term Results of Cell-Free Biodegradable Scaffolds for *In Situ* Tissue-Engineering Vasculature: In a Canine Inferior Vena Cava Model

**DOI:** 10.1371/journal.pone.0035760

**Published:** 2012-04-19

**Authors:** Goki Matsumura, Naotaka Nitta, Shojiro Matsuda, Yuki Sakamoto, Noriko Isayama, Kenji Yamazaki, Yoshito Ikada

**Affiliations:** 1 Cardiovascular Surgery, The Heart Institute of Japan, Tokyo Women's Medical University, Shinjuku, Tokyo, Japan; 2 Biomedical Sensing and Imaging Group, Human Technology Research Institute, National Institute of Advanced Industrial Science and Technology, Tsukuba, Ibaraki, Japan; 3 Research and Development Department, Gunze Ltd., Ayabe-shi, Kyoto, Japan; 4 Department of Indoor Environmental Medicine, Nara Medical University, Kashihara, Nara, Japan; Sapienza University of Rome, Italy

## Abstract

We have developed a new biodegradable scaffold that does not require any cell seeding to create an *in-situ* tissue-engineering vasculature (*i*TEV). Animal experiments were conducted to test its characteristics and long-term efficacy. An 8-mm tubular biodegradable scaffold, consisting of polyglycolide knitted fibers and an L-lactide and ε-caprolactone copolymer sponge with outer glycolide and ε-caprolactone copolymer monofilament reinforcement, was implanted into the inferior vena cava (IVC) of 13 canines. All the animals remained alive without any major complications until euthanasia. The utility of the *i*TEV was evaluated from 1 to 24 months postoperatively. The elastic modulus of the *i*TEV determined by an intravascular ultrasound imaging system was about 90% of the native IVC after 1 month. Angiography of the *i*TEV after 2 years showed a well-formed vasculature without marked stenosis or thrombosis with a mean pressure gradient of 0.51±0.19 mmHg. The length of the *i*TEV at 2 years had increased by 0.48±0.15 cm compared with the length of the original scaffold (2–3 cm). Histological examinations revealed a well-formed vessel-like vasculature without calcification. Biochemical analyses showed no significant differences in the hydroxyproline, elastin, and calcium contents compared with the native IVC. We concluded that the findings shown above provide direct evidence that the new scaffold can be useful for cell-free tissue-engineering of vasculature. The long-term results revealed that the *i*TEV was of good quality and had adapted its shape to the needs of the living body. Therefore, this scaffold would be applicable for pediatric cardiovascular surgery involving biocompatible materials.

## Introduction

The use of foreign materials is necessary to repair complex heart defects. However, the materials that are commonly used are not biocompatible with the host tissue and do not have the ability to change their shape as the host grows. In addition, long-term studies of the efficacy of these materials have revealed several material-related failures, such as stenosis, thromboembolization, and calcium deposition. To solve these problems and to improve the treatment of children who require implantation of materials possessing growth potential, we have been pursuing the development of optimal biocompatible materials.

We previously reported the advantages of using biodegradable scaffolds seeded with autologous cells as tissue-engineered vascular autografts (TEVAs) in canine models [1], [2], [3] and in a human clinical study [1], [4], [5]. The key benefit of utilizing such scaffolds is that the scaffold degrades *in vivo*, thereby avoiding the long-term presence of foreign materials, while the seeded cells proliferate to form new tissue [2], [6]. We also reported that the seeded cells are able to differentiate into cells that compose the vessel wall and may act as cytokine producers [6]. However, the contributions of the seeded cells remain uncertain [6]. There was no graft-related mortality and no evidence of aneurysm formation, graft rupture, graft infection, or ectopic calcification in the late-term results for TEVAs in 25 patients (mean follow-up, 5.8 years) [4]. However, 4 of the 25 patients had graft stenosis and underwent successful percutaneous angioplasty [4]. To overcome the graft stenosis of TEVAs, we explored a new scaffold without cell seeding, and achieved acceptable long-term results as evaluated by new techniques involving an intravascular ultrasound imaging system. This new scaffold can be implanted by a simple cost-effective procedure, because no cell preparation and seeding are necessary.

In the present study, we evaluated the long-term outcomes, usefulness, and basic characteristics of an *in-situ* tissue-engineering vasculature (*i*TEV) constructed through cell-free and direct implantation of the new biodegradable scaffold in a canine model.

## Results

### Overview, the mechanical properties and degradation of a newly developed biodegradable scaffold

**Figure 1:**
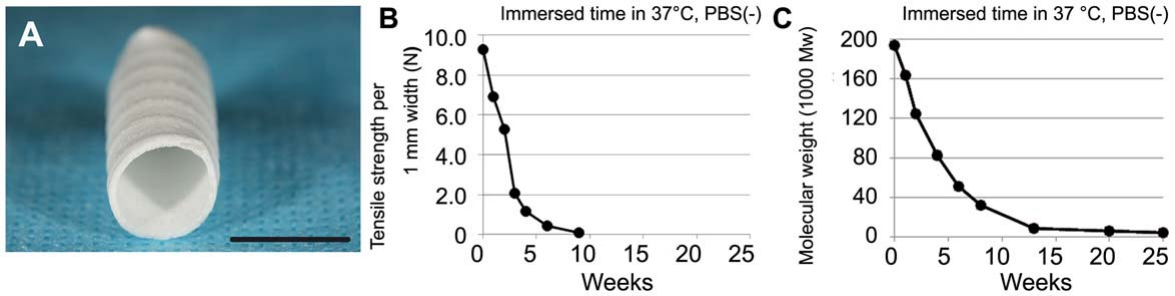
Overview of the new biodegradable scaffold and its degradation. A, biodegradable scaffold 8 mm in diameter; bar, 1 cm. B, tensile strength changes in biodegradable scaffolds *in vitro*; scaffold strength diminished remarkably over 4 weeks. C, Mw changes in scaffold immersed in 37°C PBS; gradual hydrolysis decreased Mw.

Shown as an overview in Figure 1A, this scaffold degraded by hydrolysis after implantation. Changes over time in the scaffold's mechanical strength and molecular weight in vivo are shown in Figures 1B and 1C. Most of the scaffold's strength was lost within a month and molecular weight decreased within 6 months, suggesting that the scaffold was almost entirely degraded and resorbed into the body during the 6-month implantation.

### Biomechanical changes of biodegradable scaffolds

The mechanical properties of biodegradable scaffolds *in vitro* were evaluated by soaking in phosphate buffered saline (PBS) at 37°C and testing with a tensiometer, and it was found that the scaffold degraded by hydrolysis, losing half of its tensile strength in 2–3 weeks and entirely within 10 weeks. The scaffold's tensile strength per cm width at 0, 1, 2, 3, 4, 6, and 9 weeks *in vitro* were 9.3, 6.9, 5.3, 2.1, 1.2, 0.4, and 0.1 N, respectively (Fig. 1B). The scaffold's molecular weight changes under these conditions decreased with time, decreasing by 0, 1, 2, 4, 6, 8, 13, 20, and 25 weeks to 194267, 163566, 124383, 82709, 51737, 32867, 8655, 6190, and 4387 daltons, respectively (Fig. 1C).

### Macroscopic and histological findings of *i*TEV

**Figure 2:**
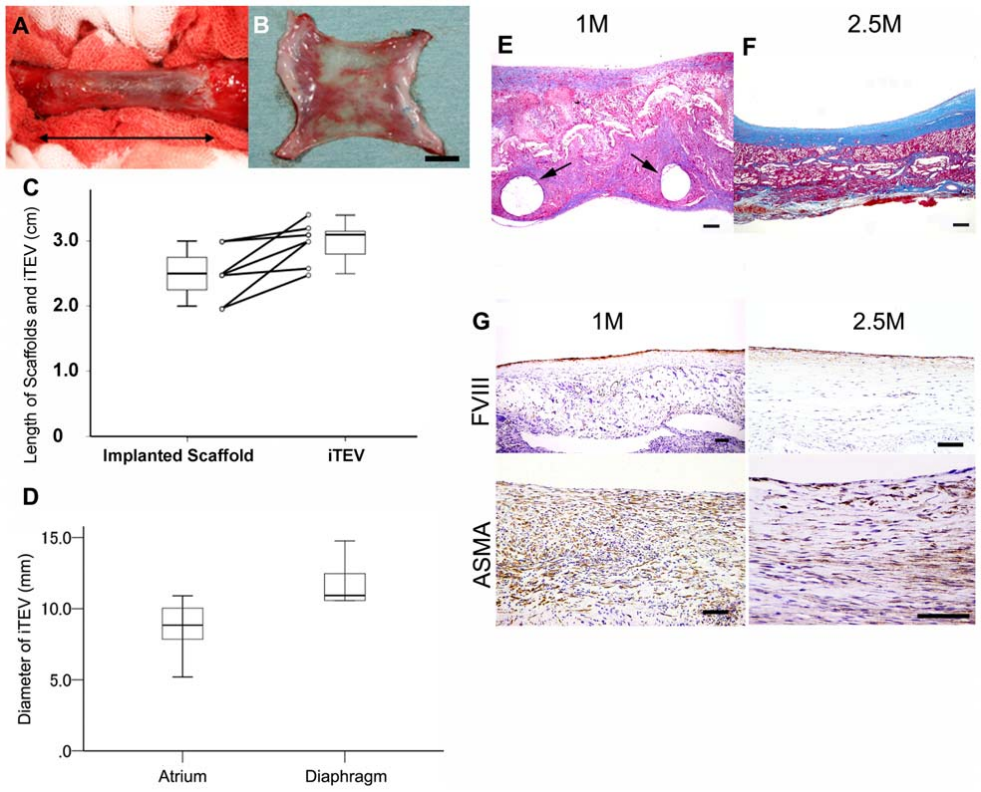
Macroscopic views, changes of length, diameter, and histological findings of *i*TEV. A, macroscopic view of *i*TEV appearance at 2 years when the dog's chest was reopened; black double-headed arrow, distance between two *i*TEV suture lines, representing *i*TEV length. B, macroscopic view of *i*TEV internal surface; smooth endothelialized surface and thin vessel wall; bar, 1 cm. C, implanted scaffold lengths and explanted *i*TEVs (n = 7) at 2 years after implantation; scaffold length data when implanted and *i*TEVs when explanted expressed as box-whisker plot; each individual length difference expressed as dot-to-dot. D, *i*TEV diameters at sites close to diaphragm and right atrium (n = 7) at 2 years after implantation; lines, lower, median, and upper quartile values; whiskers, extent of remaining data. E, *i*TEV histology at 1 month (Masson's trichrome staining); black arrows indicate P(GA/CL) monofilament remaining at 1 month after implantation; bar, 100 µm. F, iTEV histology at 2.5 months; P(GA/CL) monofilament no longer present; bar, 100 µm. G, factor VIII positive and ASMA-positive cells expressed in *i*TEV at 1 and 2.5 months; bar, 100 µm.

**Figure 3:**
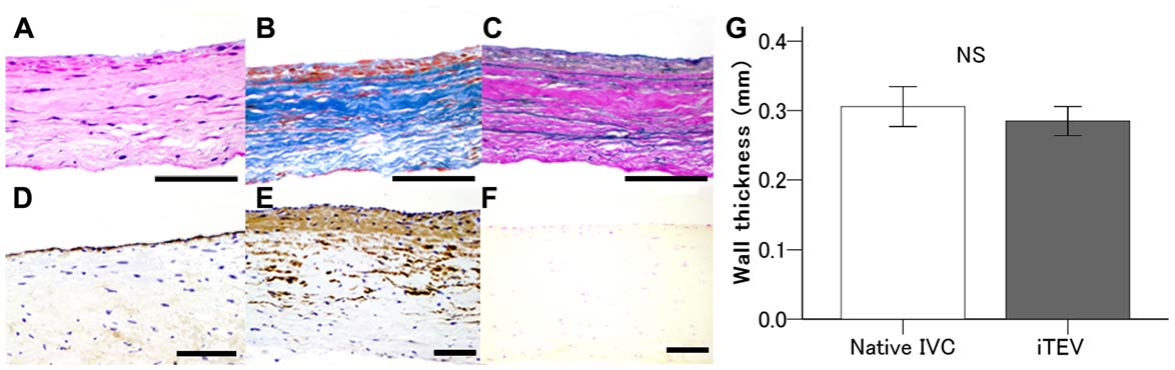
Histological results of an *i*TEV at 2 years. Representative *i*TEV histological sections: A, H&E staining, flattened cell monolayer lining *i*TEV surface layer of endothelial cells. B, Masson's trichrome staining, smooth muscle cells (red) and collagen fibers (blue). C, Victoria blue–van Gieson staining, elastic fiber and smooth muscle cell proliferation; D and E, factor VIII and ASMA clearly visible, respectively. F, modified von Kossa staining, no calcified lesions; bars, 100 µm. G, wall thickness of native IVC and *i*TEV at 2 yr (*n* = 7 each) in histological samples; 10 sites for each sample; no significant difference between wall thicknesses of native IVC and *i*TEV (*p* = 0.511 by the Mann–Whitney U test); NS, not significant.

All animals remained alive and well without any serious complications until euthanasia at 1(n = 3), 2.5(n = 3), and 24 months (n = 7). Vessel-like *i*TEVs were recovered at 2 years by dissection and examined (Fig. 2A, black arrow, distance between suture sites at implantation). Internal *i*TEV sections showed completely endothelialized surfaces and thin vessel walls (Fig. 2B). Implanted scaffold lengths and *i*TEVs after explantation from individual dogs showed that all *i*TEVs increased in length over the 2-year period, ranging from 0.1 to 1.0 cm (mean ±SEM, 0.48±0.15 cm, Fig. 2C). When explanted at 2 years, the mean diameters of *i*TEVs sited close to the diaphragm and the atrium were 11.8±0.7 and 8.7±0.8 mm, respectively (Fig. 2D). All *i*TEV diameters increased during the 2-year period compared with the initial implanted 8 mm tubular-shaped scaffold. The *i*TEV sections at 1 month showed the presence of P(GA/CL) monofilaments (Fig. 2E), but they appeared to be resorbed in *i*TEV sections at 2.5 months (Fig. 2F). Immunohistological study revealed endothelialization (factor VIII-positive) and smooth muscle cell (alpha smooth muscle cell actin [ASMA]-positive) proliferation in *i*TEV sections at 1 and 2.5 months (Fig.2G). The components of the four basic vascular layers, endothelial cells (Factor VIII-positive), smooth muscle cells (ASMA-positive), elastic fibers, and collagen fibers, were observed in *i*TEV sections at 2 years (Fig. 3A–E); no calcified lesions were observed (Fig. 3F). As the biodegradable materials were entirely degraded within 6 months, these findings suggested that the application of P(GA/CL) monofilaments to the scaffold did not cause calcification. A comparison of the wall thicknesses of native inferior vena cava (IVC) and *i*TEV showed that native IVC and *i*TEV walls were 0.31±0.01 and 0.29±0.01 mm thick, respectively, and were not significantly different (*p* = 0.511, Fig. 3G).

### Angiographic and biomechanical findings using the IVUS

**Figure 4:**
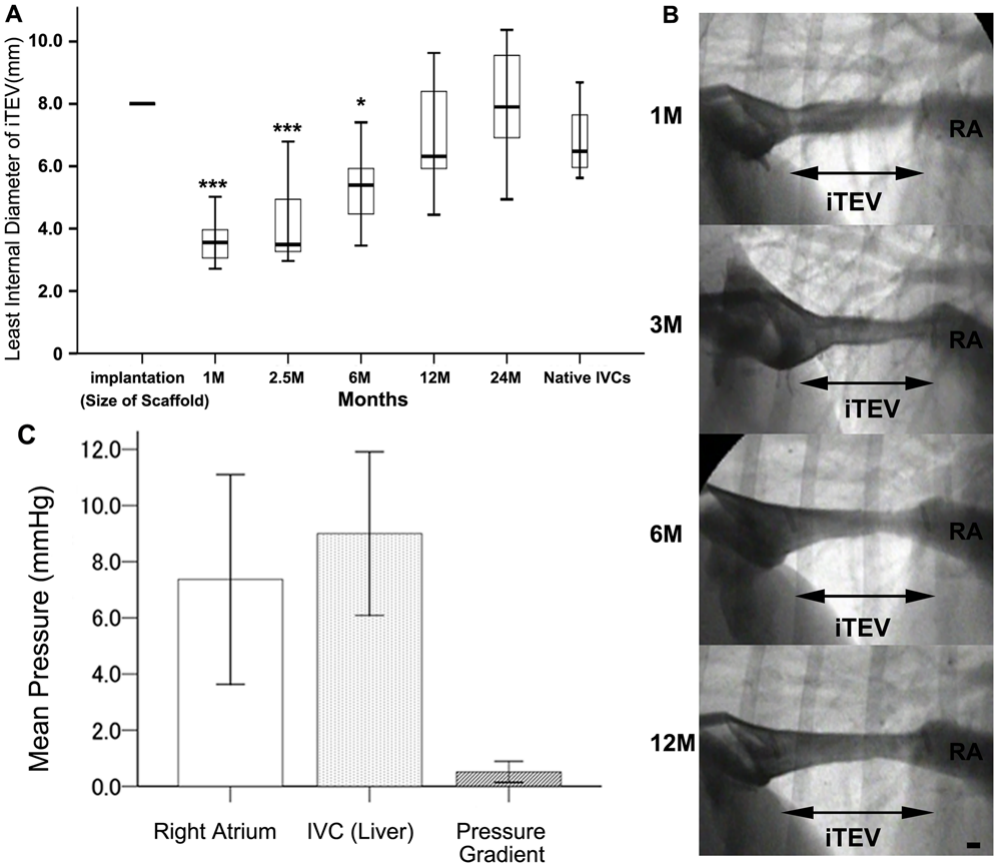
Changes of diameters, angiographic findings, and pressure gradient of *i*TEVs. A, smallest *i*TEV diameter changes from angiographies at 1, 2.5, 6, 12, and 24 months after implantation; ***, p<0.001 and *, p<0.05 compared to native IVCs by the Mann–Whitney U test. B, representative *i*TEV angiographies at 1, 2.5, 6, and 12 months after implantation; *i*TEV feature differences showing remodeling and tissue generation; magnification ratio adjusted using sheath size; bar, 7Fr, ∼2.33 mm; RA, right atrium. C, mean pressures at right atrium and distal liver (IVC); mean pressure gradient between two sites (*n* = 7 each); pressure gradient between *i*TEV proximal and distal sites, 0.51±0.19 mmHg, when measured by Millar pressure catheter system.

Seven animals were evaluated by angiography while in a right lateral position and an intravascular ultrasound imaging system (IVUS) employed in a catheter laboratory to confirm the *i*TEV characteristics, pressure gradient, and elastic modulus. Time-course changes of the smallest internal diameters of *iTEVs* measured by angiography revealed that the *i*TEV diameters at 1, 2.5, 6, 12, and 24 months were 3.55±0.62, 4.06±1.12, 5.28±1.22, 6.92±1.75, and 7.98±1.86 mm, respectively (Fig. 4A). The smallest internal diameters *i*TEVs increased the most in diameter during the 2-year period (Kruskal–Walis tests; *p*<0.001). It should be noted that diameter decreased during neointimal growth on the scaffold, the tissue remodeled within 6 months into native tissues with concurrent material degradation, and the internal diameter tended to increase and adapt its shape as the living body required. The smallest internal diameter of the dog native IVCs (6.81±0.52; n = 6) showed remarkable differences from those of the 1, 2.5, and 6-month *i*TEV, but no significant differences from the 12 and 24-month *i*TEVs (Mann–Whitney U test; native IVCs versus 1- or 2.5-month *i*TEVs, *p*<0.001, native IVCs versus 6-month iTEVs, *p*<0.05). Angiogram data showed some feature changes during the *i*TEV remodeling and scaffold degradation processes (Fig. 4B), and following the angiograms, pressure studies were performed using a Millar pressure catheter system, which showed that the pressure gradient between the *i*TEV proximal and distal sites was 0.51±0.19 mmHg at 24 months (Fig. 4C).

**Figure 5:**
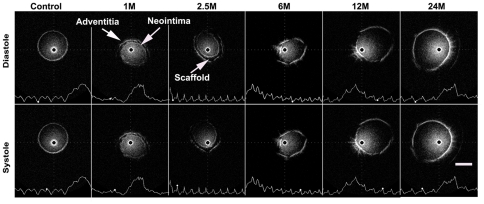
IVUS images of an *i*TEV. Representative IVUS images of *i*TEV in diastole and systole phases taken immediately after implantation (control) and at 1, 2.5, 6, 12, and 24 months; showing *i*TEV wall thickness and internal diameter changes and scaffold degradation; waves, venous pressure measured by Millar pressure catheter system; white dots on waves, phase of venous pressure and *i*TEV wall motion; bar, 5 mm.

IVUS images showing *i*TEV changes over time indicated that the scaffold was located between the neointima and adventitia during tissue development (Fig. 5, 1, and 2.5 months).

**Figure 6:**
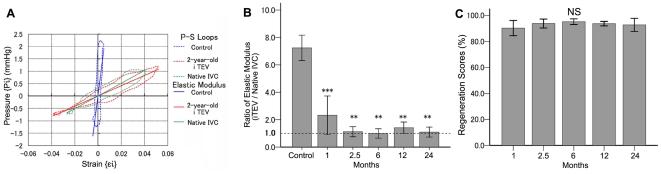
Biomechanical analyses of IVC and *i*TEV at 2 years. A, representative P–S loop data for control scaffold and *i*TEV and native IVC at 2 yr; P–S loops from time-dependent strain and pressure measurements 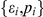, used to calculate sample elastic moduli. B, elastic moduli ratios of *i*TEV to native IVC at 0 (control), 1, 2.5, 6, 12, and 24 months (n = 7); significant differences between control and samples (*p*<0.0001 by ANOVA); ***, *p*<0.001, control vs. 1 month and **, *p*<0.01, control vs. 2.5–24 months by Dunnett's *post hoc* test. C, *i*TEV RS at 1, 2.5, 6, 12, and 24 months (n = 7); RS maintained by scaffold while tissue in infancy; data show *i*TEV elasticity not affected by time after implantation; Note, P(GA/CL) monofilament and P(LA/CL) sponge scaffold lose strength within ∼2.5 and 6 months, respectively (*p* = 0.443 by ANOVA); NS, not significant.

Data for the pressure-to-strain (P-S) loops of the *i*TEV and native IVC at 2 years and of a control (scaffold itself) showed that the ratios of the elastic modulus of the *i*TEV to native IVC at 0 (control), 1, 2.5, 6, 12, and 24 months were 7.3±0.5, 2.3±0.7, 1.1±0.2, 1.0±0.2, 1.4±0.2, and 1.1±0.2, respectively (Fig. 6). The elastic modulus of the *i*TEV decreased over time and was almost the same as the native IVC after 2.5 months. Also, there were significant differences between the control scaffold and the other samples, and no significant differences among the samples at 1, 2.5, 6, 12, and 24 months.

Finally, elastic moduli data were used to calculate *i*TEV regeneration scores at 1, 2.5, 6, 12, and 24 months and found to be 90.3±2.9%, 93.8±1.7%, 95.8±1.1%, 93.7±0.8%, and 92.7±2.5%, respectively (Fig. 6C). There were no significant differences among these samples (*p* = 0.836).

### Biochemical findings

**Figure 7:**
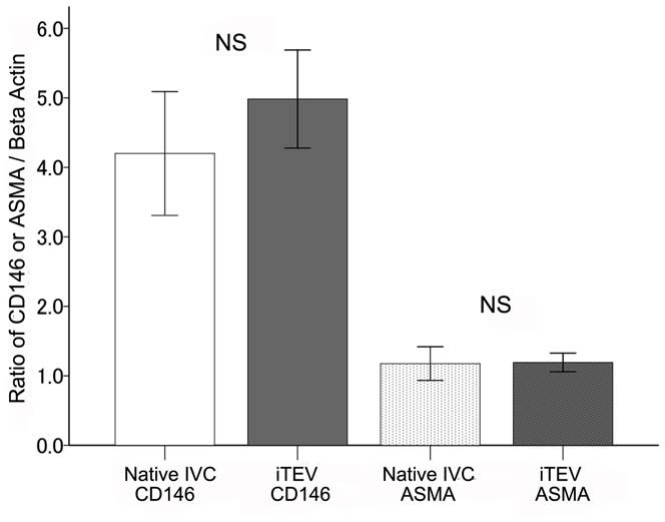
Protein densitometry of endothelial cells and smooth muscle cells of the *i*TEV and IVC. Bar graphs of CD146 and ASMA protein densitometry normalized by beta-actin concentrations; all results from native IVC and *i*TEV samples at 2 years (*n* = 7 each); ratios of ASMA and CD146 to beta-actin, means ±SEM; no significant differences (CD146, *p* = 0.193 and ASMA, *p* = 0.907 by unpaired Student's *t*-test); NS, not significant.

There were no significant differences in the protein content of *i*TEVs and native IVCs at 2 years, with the densitometry ratios of CD146 protein to beta-actin in native IVCs and *i*TEVs at 4.2±0.4 and 5.0±0.4, respectively (*p* = 0.193) and the densitometry ratios of ASMA protein to beta-actin in IVCs and *i*TEVs at 1.1±0.1 and 1.2±0.1, respectively (*p* = 0.907, Fig. 7).

**Figure 8:**
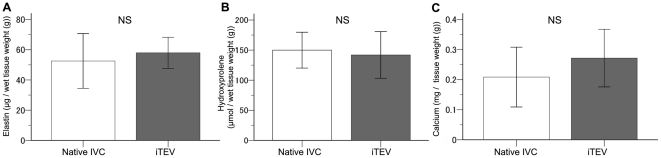
Biochemical analyses of the *i*TEV and IVC at 2 years. A–C, elastin, hydroxyproline, and calcium contents in IVC and *i*TEV at 2 years; data, means ±SEM (*n* = 7 each); no significant differences (elastin, *p* = 0.615 and hydroxyproline, *p* = 0.750 by Student's *t*-test; calcium, *p* = 0.165 by the Mann–Whitney U test); NS, not significant.

There were also no significant differences in the elastin, hydroxyproline, and calcium contents between normal IVCs and *i*TEVs (Fig. 8A–C), with elastin content of native IVCs and *i*TEVs at 52.6±9.1 and 57.9±5.1 µg/g wet tissue weight, respectively (*p* = 0.615). Based on hydroxyproline concentration, the collagen concentrations of native IVCs and *i*TEVs were 150±15 and 142±19 µmol/g wet tissue weight, respectively (*p* = 0.750). And finally, the calcium content of native IVCs and *i*TEVs were 0.21±0.05 and 0.27±0.05 µg/g wet tissue weight, respectively (*p* = 0.165).

## Discussion

In the present study, evaluation of the long-term results from *i*TEVs, produced by tubular composite scaffold implantation without cell seeding in a canine model, revealed their efficacy in terms of patency and in their histological, biomechanical, and biochemical similarities to native vessels. It was clearly demonstrated that the morphology of the implanted biodegradable scaffold was remodeled.

Previously here, clinical applications of tissue-engineered vascular autographs (TEVAs) have been successful, using cultured venous cell mixtures as a cell source with seeding onto a biodegradable scaffold prior to implantation [1]. However, this protocol proved difficult to manipulate, as bovine serum was necessary and the cell preparation process took several weeks. Thus, the procedure was altered to incorporate mononuclear bone marrow cells (MN-BMCs) as the cell source, as they can be easily prepared on the day of procedure [1]. MN-BMC-seeded TEVAs have regenerated vessel-like structures with good results in animal studies and human clinical trials [1], [2], [4]–[6], [8]. However, this protocol still requires complex cell preparation processes [9], which represents a critical barrier for broad clinical applications. Consequently, development of a scaffold for tissue engineering of vasculature that does not require cell seeding is in development here.

Although various types of scaffolds have been examined in this laboratory, successful patency rates did not differ between scaffolds with or without cell seeding (unpublished observations). Histological observations in a previous study here suggested that tissue regeneration was chiefly dependent on neighboring cells and not on seeded cells [6]. Furthermore, seeded MN-BMCs contributed as producers of cytokines, which induce cell recruitment for constructing mature tissues, rather than differentiating into mature vasculature components [10]. Therefore, it was concluded that seeding of autologous cells onto a scaffold is not a promising strategy and that it might be possible to create an *i*TEV with an optimized biodegradable scaffold without cell seeding.

Although the late-term results of TEVAs are acceptable, the problem of graft stenosis generation in some patients still exists [4]. Such stenotic changes may cause tissue regeneration disorders, such as blood flow disturbance, thrombogenesis, or graft occlusion. Graft stenosis was also found to occur within a few months after implantation and a graft was never occluded or suffered from stenosis after complete endothelial coverage (unpublished observations), suggesting that scaffold reinforcement during the relatively early period of tissue development is essential.

The focus here was on the design of a biodegradable scaffold with optimal mechanical properties and degradation time. It should maintain its shape during early endothelialization after implantation to avoid blood flow disturbances and overgrowth of fibrotic tissue. From previous studies here, neointimal growth with internal surface endothelialization of the scaffold occurs within a month after implantation (unpublished data). During this initial period, if fibrotic tissues massively proliferated, stenotic changes in the *i*TEV would have been observed (unpublished observations). Thus, a material that maintains its shape during the endothelialization period is considered able to avoid graft stenosis and a novel biodegradable scaffold was developed that was reinforced with P(GA/CL) monofilaments on the outer surface, to maintain *i*TEV shape for the initial crucial period following implantation. These monofilaments, however, decreased in strength within a month and then degraded within 2 months, with the P(LA/CL) spongy scaffold concurrently but more slowly degrading in the *i*TEV to create a relatively constant elasticity in the *i*TEV during the tissue-remodeling process. As a result, this new P(GA/CL) monofilament-reinforced scaffold prevented graft stenosis and allowed complete endothelialization of the graft inner surface. Furthermore, these *i*TEVs reached optimal elasticity after a month, judged from RS values. Consequently, tissue regeneration was also attained without complications, resulting in long-term *i*TEV patency. These findings suggested that this new biodegradable scaffold possessed significant potential for reducing the incidence of graft stenosis observed in previous clinical trials.

As the mechanical properties of a scaffold are closely related to biocompatibility, maturity, and structural integrity of grafted materials, the *i*TEV elastic modulus has been evaluated [11], [12]. Although mechanical tests of the excised tissue samples from euthanized animals indicated the immediate *i*TEV character, it has been impossible to determine the course of continuous changes in the elastic modulus *in vivo* [2]. In the present study, a novel *in vivo* system for tracking elastic moduli is proposed which uses an IVUS and a pressure catheter for evaluating the *i*TEV mechanical properties [7]. This technique enabled assessment of *i*TEV elasticity changes as well as reduction of the number of animals required. The chosen scaffold degraded and changed its elastic moduli during tissue development and regeneration to form mature vasculature, such that, at 1–2.5 months, when the tissue was still in its infancy, *i*TEVs revealed adequate RSs and elastic moduli ratios. At 1–2 years, after scaffolds resorption, *i*TEVs showed RSs and elastic moduli ratios similar to native IVCs, which suggested significant remodeling of *i*TEVs.

The IVUS images showed changes in *i*TEV wall characteristics *in vivo*, in which neointima developed on the scaffold interior surfaces, the internal diameter reduced in the early phase, and the internal diameter increased, during which time the scaffold was resorbed and tissue developed.

It is widely known that artificial grafts are reliable, stable, and in common use throughout the world. However, concerns still remain regarding non-biocompatibility, such as calcification [13], thrombosis [14], [15], [16], and decreased diameter due to pseudo-intimal peel formation [16], [17], [18]. Although an *i*TEV is initially an artificial material when implanted, the scaffold resorbs over time, allowing regeneration of “native vessels."

The *i*TEV is a promising alternative to artificial grafts, particularly in venous positions, and have benefits, such as avoidance of anticoagulation therapy and reductions in the incidence of thromboembolic complications and calcification [2], [6], and the potential to remodel into an appropriate size in response to vessel flow or a patient's growth [5]. Thus, in the field of congenital heart diseases, this novel strategy could provide great benefits for children who require anatomical repair using an artificial graft. Surgeons tend to implant oversized grafts in pediatric patients, increasing the chance of thrombosis in the prosthetic graft. Therefore, an ideal graft that adapts to the child's growth is desired, as more suitable future-sized grafts cannot be implanted in pediatric patients. The present findings have shown that an *i*TEV undergoes remodeling to meet the body's requirements, evidenced by changes in *i*TEV diameter and length over time. Furthermore, *i*TEVs managed to maintain their original features and mechanical properties, while avoiding stenosis and calcification. All these findings supported the realistic possibility of applying *i*TEVs to pediatric cardiovascular surgery. At present, iTEVs are envisioned for use in systemic venous reconstruction, such as modified Fontan operations (extracardiac total cavo-pulmonary connection) and modified Warden procedures for partial anomalous pulmonary venous connection. These *i*TEVs are biocompatible, anti-thrombogenic, and have the remodeling potential required by pediatric patients with congenital heart diseases as they grow. For extended applications of iTEVs, further studies of the scaffold under high-pressure circumstances should be conducted.

In conclusion, an *i*TEV with good long-term results can be constructed by direct implantation of this new biodegradable scaffold. As the scaffold degraded and autologous tissue developed *in vivo*, the new “regenerated", “reconstructed", and “adapted" vasculature possessed biocompatible characteristics, thereby avoiding unwanted calcification. Based on the present findings, the protocol for *i*TEV development can be simplified and made more versatile. We think that this novel “Cell-Free Tissue Engineering for Vasculature" can easily be applied to treatments of patients who require surgical interventions with artificial grafts to provide them a better quality of life.

## Materials and Methods

### Ethics statement

The ethical committee of Tokyo Women's Medical University reviewed and approved the study protocol (Permit Number: 07-68).

### Newly developed biodegradable scaffold for cell-free *i*TEV

A composite tubular scaffold has been previously developed, consisting of polyglycolide (PGA) knitted fibers and an L-lactide and ε-caprolactone copolymer (P(LA/CL)) sponge with outer glycolide and ε-caprolactone copolymer (P(GA/CL)) monofilament reinforcement. The PGA fibers and P(LA/CL) sponge were the same as those used in previous studies to create TEVAs [1], [2]. The P(GA/CL) monofilament (0.45 mm diameter) was wound around the scaffold outer surface with a pitch of 3 mm (Fig. 1A). As the P(GA/CL) monofilament will lose its strength within 2 months owing to non-enzymatic hydrolysis, it was used to provide reinforcement during the crucial tissue construction period. The new scaffold possessed 27 times higher compression strength than previous scaffolds and was sterilized with ethylene oxide gas prior to implantation.

### Mechanical and degradation tests of biodegradable scaffolds *in vitro*

Biodegradable scaffolds were cut in ∼1-cm width rings and soaked in PBS at 37°C for 0–25 weeks. For assessment, the tensile strength (N) of sample scaffolds at 0–9 weeks were measured on a tensiometer (EZTest, Shimadzu, Kyoto, Japan) at a crosshead speed of 50 mm/min. The molecular weight (Mw) was determined by gel permeation chromatography using a Shimadzu GPC-System equipped with a pump, degasser (LC-10AT VP) at 1.0 ml/min and a refractive index detector (RID-10A, Shimadzu). The column was eluted with trichloromethane at 1.0 ml/min at 40°C and calibrated with polystyrene standards over an Mw range of 4,000–1,600,000 daltons.

### Animal experiments

Thirteen healthy adult female beagles (NARC, Tomisato, Japan) with a mean weight of 9.3 kg (7.0–11.5 kg) were obtained for this study, with six animals euthanized for histological examinations at 1 (n = 3) and 3 (n = 3) months, and seven animals euthanized at 24 months for histological and biochemical analyses. For surgeries, animals were anesthetized with pentobarbital (1 mg/kg body weight) and atropine sulfate (0.08 mg/kg body weight), with heparin (500 U/kg body weight) administered intravenously for anticoagulation during anastomoses. Each scaffold (8 mm diameter and 2–3 cm long) was implanted into the IVC, as described previously [2], [6], and the animals followed by IVUS until 24 months (1 (n = 11), 2.5 (n = 11), 6 (n = 8), 12 (n = 7), and 24 (n = 7) months). Aspirin (2 mg) was orally administered for the first month after surgery as an anticoagulation therapy and subsequently maintained without anticoagulants until euthanasia.

After dissection, the *i*TEV length was measured directly between the two suture lines and the *i*TEV then longitudinally dissected to allow histological and biochemical analyses. For each assay, samples of native IVC and *i*TEV were dissected, rinsed with PBS, and stored at −20°C until analysis.

### Histological examination

Longitudinally incised *i*TEV and native IVC samples as controls were fixed in 4% paraformaldehyde in pH 7.0 PBS, embedded in paraffin, and sectioned at 4–5 µm. Some sections were subjected to hematoxylin and eosin (H&E), Masson's trichrome, Victoria blue–van Gieson, or modified von Kossa staining, as previously described [2], [6]. Immunostaining of other sections was performed with antibodies against factor VIII (1∶1000) and ASMA (clone 1A4; 1∶1000; Dako Japan Inc., Tokyo, Japan). *i*TEV wall thicknesses were measured at 10 different sites in these sections and all histological examinations and measurements performed using a microscope (Biozero BZ-8000; Keyence Corp., Osaka, Japan) and accompanying analytical software (BZ-Analyzer; Keyence Corp.).

### Angiography and biomechanical analyses using IVUS

Subject animals were anesthetized using the protocol described above and placed in sterilized conditions for femoral vein dissection. An 8-Fr long catheter sheath (Terumo Corp., Tokyo, Japan) was placed through the femoral vein adjacent to the IVC, close to the diaphragm. An X-ray fluoroscope was used to identify sheath and catheter locations and for digital angiography. Approximately 5 ml of angiographic agent was injected to evaluate *i*TEV features and to measure the smallest *i*TEV internal diameter during the regeneration process and in native IVCs of control dogs. Next, a 2-Fr Millar Mikro-Tip catheter pressure transducer (Millar Instruments Inc., Houston, TX) and a 2.5-Fr IVUS (Terumo Corp.) were inserted through the long catheter sheath. Simultaneously, a Millar Mikro-Tip catheter was used to measure the pressure gradient between *i*TEV proximal and distal sites.

The elastic moduli of *i*TEVs and native IVCs were analyzed based on the P-S loops obtained using synchronized ultrasonic B-mode images and pressure signals recorded in the lumen, acquired using ultrasound and pressure sensor catheters [7]. Vessel wall deformation induced by circumferential stress was analyzed using successive ultrasonic B-mode images reconstructed using RF data. Repeated imaging of the lumen on ultrasonic B-mode images enabled calculation of the time-dependent luminal circumferential length 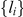 (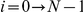; 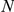, number of B-mode images). When the 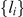 in the initial frame is expressed by {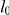}, the circumferential strain 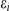 on the 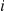-th frame can be calculated as follows:
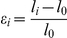



Plotting of the time-dependent pressure {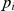} and circumferential strain {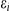} values allowed calculation of the P-S loop and the dynamics of the *i*TEV wall and native IVC. The elastic modulus (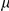) was calculated using the measured parameters that make up the P-S loop 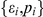 as follows:
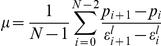



Using the elastic moduli of the *i*TEVs and IVCs, the RS was calculated by comparing the elastic moduli of native IVCs with that of *i*TEVs using the following formula, which predicts the degree of *i*TEV regeneration:
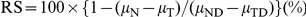
where 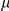
_N_ and 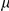
_T_ indicate the elastic moduli of native IVC and *i*TEV at any time, respectively, and 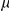
_N0_ and 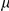
_T0_ indicate the elastic moduli of native IVC and *i*TEV immediately after implantation, respectively. In other words, the difference between the elastic moduli of *i*TEV and native IVC at any time was normalized by that observed immediately after implantation. Based on this formula, RSs of 0% and 100% indicated incomplete and complete regeneration, respectively.

### Biochemical analyses of protein, elastin, hydroxyproline, and calcium content

After thawing, *i*TEV and native IVC samples were weighed and homogenized at a 1/20 (w/v) ratio of tissue to Tissue Protein Extraction Reagent (Thermo Fisher Scientific Inc., Rockford, IL) for protein extraction, and the total protein content determined using the Bradford assay. Samples of the denatured proteins, at 15 µg per lane, were separated in 4–12% polyacrylamide gels (NuPAGER Novex® Bis-Tris [Bis (2-hydroxyethyl) imino-Tris (hydroxymethyl) methane-HCl] Midi Gels; Invitrogen, Carlsbad, CA) and transferred to polyvinylidene difluoride membranes using an iBLOT dry blotting system (Invitrogen). As primary antibodies, an anti-ASMA antibody (1∶1000, clone 1A4; Dako) and an anti-CD146 antibody (1∶1000; Epitomics, Inc., Burlingame, CA) were used to detect endothelial cell proteins in *i*TEVs, with an anti-human beta-actin antibody (1∶2000; Abcam, Cambridge, MA) used as an internal control. A WesternBreeze® Chemiluminescence Immunodetection Kit and a BenchPro™ 4100 system (Invitrogen) were used for detection of antigen-antibody complexes immobilized on polyvinylidene difluoride membranes, according to the manufacturer's protocol. After membrane enhancement by treatment with the kit chemiluminescent reagent, chemiluminescence images were acquired using a cooled CCD camera (LAS-3000 Mini; Fujifilm Corp., Tokyo, Japan) and analyzed using image analysis software (MultiGauge; Fujifilm Corp.).

A commercially available elastin assay kit (Fastin Elastin Assay Kit; Biocolor, Ltd., Belfast, Northern Ireland) was used to quantify the elastin content of *i*TEV and native IVC samples. Insoluble tissue elastin was solubilized by hot oxalic acid treatment, precipitated, and mixed with the Fastin dye reagent. The elastin–dye complex was collected by centrifugation, dye bound to the elastin pellet solubilized with the Destain reagent, and the recovered dye concentration measured at 513 nm.

Sample hydroxyproline contents were measured by high-performance liquid chromatography (HPLC). In preparation, tissue samples were weighed and digested with 12 N HCl for 24 h, the resulting suspensions hydrolyzed with 0.5 ml of 12 N HCl for 20 h at 100°C, and after neutralization and centrifugation, a 0.1-ml aliquot of each supernatant was mixed with 1.5 ml of 0.3 N lithium hydroxide solution for analysis by HPLC.

The calcium concentrations of the tissue samples were determined using a Zeeman polarized atomic absorption spectrophotometer (Model Z-6100; Hitachi Co., Ltd., Tokyo, Japan). Tissue samples were weighed and digested with nitric acid and concentrated hydrogen peroxide (ratio, 4/1) and a model MLS1200 microwave system (Milestone Inc., Monroe, CT) was used to achieve sample decomposition. Subsequently, 25 µl of each sample was added to 2 ml of 10% lanthanum chloride and the calcium content measured by reference to a standard calcium solution (Wako Pure Chemical Industries, Ltd., Osaka, Japan).

### Statistical analysis

An unpaired Student's *t*-test or the Mann–Whitney U test was used to compare the results from the control and time groups depending on each group's data variance. One-way analysis of variance (ANOVA) followed by Dunnett's *post hoc* analyses or the Kruskal–Wallis test were used when the variances were not appropriate. All data were expressed as means ±SEM, and probability values of *p*<0.05 considered to indicate statistical significance. IBM SPSS Statistics 19 (IBM Japan, Ltd., Tokyo, Japan) was used for statistical analyses.

## References

[1] Matsumura G, Hibino N, Ikada Y, Kurosawa H, Shin'oka T (2003). Successful application of tissue engineered vascular autografts: clinical experience.. Biomaterials.

[2] Matsumura G, Ishihara Y, Miyagawa-Tomita S, Ikada Y, Matsuda S (2006). Evaluation of tissue-engineered vascular autografts.. Tissue Eng.

[3] Watanabe M, Shin'oka T, Tohyama S, Hibino N, Konuma T (2001). Tissue-engineered vascular autograft: inferior vena cava replacement in a dog model.. Tissue Eng.

[4] Hibino N, McGillicuddy E, Matsumura G, Ichihara Y, Naito Y (2010). Late-term results of tissue-engineered vascular grafts in humans.. J Thorac Cardiovasc Surg.

[5] Shin'oka T, Matsumura G, Hibino N, Naito Y, Watanabe M (2005). Midterm clinical result of tissue-engineered vascular autografts seeded with autologous bone marrow cells.. J Thorac Cardiovasc Surg.

[6] Matsumura G, Miyagawa-Tomita S, Shin'oka T, Ikada Y, Kurosawa H (2003). First evidence that bone marrow cells contribute to the construction of tissue-engineered vascular autografts in vivo.. Circulation.

[7] Nitta N, Yamane T, Matsumura G, Shiina T (2008). Ultrasonic measurement of vascular scaffold elasticity using catheter system.. Conf Proc IEEE Eng Med Biol Soc.

[8] Hibino N, Shin'oka T, Matsumura G, Ikada Y, Kurosawa H (2005). The tissue-engineered vascular graft using bone marrow without culture.. J Thorac Cardiovasc Surg.

[9] Mirensky TL, Nelson GN, Brennan MP, Roh JD, Hibino N (2009). Tissue-engineered arterial grafts: long-term results after implantation in a small animal model.. J Pediatr Surg.

[10] Roh JD, Sawh-Martinez R, Brennan MP, Jay SM, Devine L (2010). Tissue-engineered vascular grafts transform into mature blood vessels via an inflammation-mediated process of vascular remodeling.. Proc Natl Acad Sci U S A.

[11] de Korte CL, Pasterkamp G, van der Steen AF, Woutman HA, Bom N (2000). Characterization of plaque components with intravascular ultrasound elastography in human femoral and coronary arteries in vitro.. Circulation.

[12] de Korte CL, van der Steen AF, Cespedes EI, Pasterkamp G (1998). Intravascular ultrasound elastography in human arteries: initial experience in vitro.. Ultrasound Med Biol.

[13] Hayabuchi Y, Mori K, Kitagawa T, Sakata M, Kagami S (2007). Polytetrafluoroethylene graft calcification in patients with surgically repaired congenital heart disease: evaluation using multidetector-row computed tomography.. Am Heart J.

[14] Alexi-Meskishvili V, Ovroutski S, Ewert P, Dahnert I, Berger F (2000). Optimal conduit size for extracardiac Fontan operation.. Eur J Cardiothorac Surg.

[15] Lemler MS, Ramaciotti C, Stromberg D, Scott WA, Leonard SR (2006). The extracardiac lateral tunnel Fontan, constructed with bovine pericardium: comparison with the extracardiac conduit Fontan.. Am Heart J.

[16] Ovroutski S, Ewert P, Alexi-Meskishvili V, Stiller B, Nurnberg JH (2004). Comparison of somatic development and status of conduit after extracardiac Fontan operation in young and older children.. Eur J Cardiothorac Surg.

[17] Amodeo A, Galletti L, Marianeschi S, Picardo S, Giannico S (1997). Extracardiac Fontan operation for complex cardiac anomalies: seven years' experience.. J Thorac Cardiovasc Surg.

[18] Lee C, Lee CH, Hwang SW, Lim HG, Kim SJ (2007). Midterm follow-up of the status of Gore-Tex graft after extracardiac conduit Fontan procedure.. Eur J Cardiothorac Surg.

